# Cytochrome *c* oxidase-intermediate fibres: Importance in understanding the pathogenesis and treatment of mitochondrial myopathy

**DOI:** 10.1016/j.nmd.2012.04.003

**Published:** 2012-08

**Authors:** Julie L. Murphy, Thiloka E. Ratnaike, Ersong Shang, Gavin Falkous, Emma L. Blakely, Charlotte L. Alston, Tanja Taivassalo, Ronald G. Haller, Robert W. Taylor, Doug M. Turnbull

**Affiliations:** a Wellcome Trust Centre for Mitochondrial Research, Newcastle University, Newcastle upon Tyne, UK; b Department of Kinesiology, McGill University, and Neuromuscular Research, Montreal Neurological Institute, Montreal, Canada; c Neuromuscular Center Institute for Exercise and Environmental Medicine of Texas Health Presbyterian Hospital, Dallas, USA; d Department of Neurology, University of Texas Southwestern Medical Center, Dallas, USA

**Keywords:** Mitochondrial myopathy, mtDNA, cytochrome *c* oxidase, enzyme histochemistry

## Abstract

An important diagnostic muscle biopsy finding in patients with mitochondrial DNA disease is the presence of respiratory-chain deficient fibres. These fibres are detected as cytochrome *c* oxidase-deficient following a sequential cytochrome *c* oxidase-succinate dehydrogenase reaction, often in a mosaic pattern within a population of cytochrome *c* oxidase-normal fibres. Detailed analysis of muscle biopsies from patients with various mitochondrial DNA defects shows that a spectrum of deficiency exists, as there are a large number of fibres which do not correspond to being either completely cytochrome *c* oxidase-normal (brown staining) or cytochrome *c* oxidase-deficient (blue staining). We have used a combination of histochemical and immunocytochemical techniques to show that a population of cytochrome *c* oxidase-intermediate reacting fibres are a gradation between normal and deficient fibres. We show that cytochrome *c* oxidase-intermediate fibres also have different genetic characteristics in terms of amount of mutated and wild-type mtDNA, and as such, may represent an important transition between respiratory normal and deficient fibres. Assessing changes in intermediate fibres will be crucial to evaluating the responses to treatment and in particular to exercise training regimes in patients with mitochondrial DNA disease.

## 1 Introduction

A central finding in the investigation and diagnosis of patients with mitochondrial myopathies is the presence of respiratory deficient muscle fibres in skeletal muscle. These fibres are identified using a histochemical assay for cytochrome *c* oxidase (COX) activity, with COX-deficient fibres first identified in patients with chronic progressive ophthalmoplegia [1]. The technique was subsequently enhanced by combining the assay of COX activity with that of succinate dehydrogenase (SDH) activity [2]. The latter enzyme is fully encoded by the nuclear genome whereas the three catalytic subunits of COX are encoded by the mitochondrial genome (mtDNA) [3]. The presence of COX-deficient, but SDH-reactive or normal muscle fibres, is indicative of a defect involving mtDNA and can be primary (mt-tRNA mutations and single, large-scale mtDNA deletions), secondary changes to the mitochondrial genome (e.g. multiple mtDNA deletions or mtDNA depletion resulting from a disorder of mtDNA maintenance) or reflect a generalised mitochondrial translation deficiency. In these patients there is typically a mosaic pattern of enzyme deficiency with both COX-normal and COX-deficient fibres present in the same biopsy. Subsequent studies have extensively used this technique to explore the molecular mechanisms involved in mitochondrial myopathies, and the COX deficient fibres show high levels of mutated mtDNA (in patients with heteroplasmic mtDNA defects) associated with low levels of wild-type mtDNA [4,5].

The value of assessing COX-deficient fibres is well-established in the diagnosis of mitochondrial disease [6] but we wished to use this technique to explore the response of skeletal muscle to exercise training in patients with mitochondrial myopathies. However, when we analysed the biopsies of patients with mitochondrial myopathy undergoing resistance training we noted the presence of fibres demonstrating variation in COX activity, which we termed “COX-intermediate fibres” [7]. These intermediate-staining fibres appear to show activity levels between true COX-normal fibres and fully COX-deficient muscle fibres. Since with exercise training, any changes observed in the transition from COX-deficient to COX-normal may prove to be very subtle, it seemed highly likely that these changes with exercise would involve a fibre going through transition from COX-deficient through intermediate to COX-normal. Indeed, following a period of resistance exercise training we observed a detectable decrease in the proportion of truly COX-deficient fibres and a concomitant increase in COX-intermediate reacting fibres, although no detectable differences in the proportion of COX-normal fibres were noted [7].

In view of the potential importance of the COX-intermediate fibres in the transition from COX-normal to COX-deficient, or vice versa, we have undertaken a comprehensive study of the biochemical and molecular genetic characteristics of these fibres. We show that fibres with COX-intermediate activity are frequently observed in muscle biopsies from a range of different mitochondrial myopathies. However, since they remain a rather imprecise definition based on visualisation of the histochemical COX/SDH reaction, we wished to establish a more robust way of characterising these fibres to be able to reliably detect subtle but important muscle biopsy changes in response to disease progression or treatment. We have further assessed their characteristics using a combination of histochemical and immunohistochemical techniques and are able to show that indeed there is an observable transition between COX-normal and COX-deficient fibres, with concomitant changes in the mitochondrial genome.

## 2 Materials and methods

### 2.1 Muscle needle biopsy

Skeletal muscle samples (quadriceps) were obtained by open or needle muscle biopsy from 31 patients with mtDNA defects. The 31 patients comprised a group of 10 patients with nuclear-driven, multiple mtDNA deletions, 11 patients with single, large-scale mtDNA deletions and 10 patients with specific mt-tRNA point mutations, including the following: two patients with m.3243A>G, and single cases with mutations at m.5543T>C, m.5560G>A, m.8328G>A, m.10010T>C, m.12206T>C, m.12315G>A, m.15967G>A and m.16023G>A. Samples were frozen in isopentane cooled by liquid nitrogen for histological and histocytochemical analysis. Ethical approval was obtained from Newcastle and North Tyneside LREC.

### 2.2 Mitochondrial enzyme histochemistry

Cryostat sections (10 μm) were cut from transversely orientated muscle blocks and subjected to histochemical staining as previously described for the individual activities of COX, SDH and the sequential assay of COX/SDH activity [8]. Briefly sections were reacted for 45 min at 37 °C with COX reaction media (4 mM diaminobenzidine tetrahydrochloride, 100 μM, cytochrome *c* and 20 μg/ml catalase in 0.2 M phosphate buffer, pH 7.0) and 40 min at 37 °C with SDH media (1.5 mM nitroblue tetrazolium, 1 mM sodium azide, 200 μM phenazine methosulphate, 130 mM sodium succinate, in 0.2 M phosphate buffer, pH 7.0).

### 2.3 COX reaction rates

In order to assess if COX-normal, COX-intermediate and COX-deficient muscle fibres have different reaction rates, we measured the rates of COX activity in groups of fibres correcting for fibre type. COX reaction media was added to 10 μm thick muscle tissue sections, images were taken every 10 min for 1 h using a Zeiss Axioplan ZiE microscope with an Axiocam HRc digital camera and Axiovision image-capture software. Densitometric measurements were taken for 73 fibres from three serial sections using Zeiss KS-300 densitometry software. The densitometry scale is an inverse linear scale ranging from 0 (black) to 255 (white) [9]. The data for each fibre were combined to provide an average density for each fibre at different time points. These averages were then plotted and the reaction rate was calculated by determining the gradient of the line. The mean of all the densitometric readings for each time point for each fibre group was taken, thus providing an overall reaction rate for COX-normal, COX-deficient and COX-intermediate activities based on visualisation of the histochemical reaction.

### 2.4 Immunohistochemistry

Muscle biopsy sections (10 μm) were fixed in 4% paraformaldehyde (PFA) for 10 min at 4 °C before being permeabilised in solutions of Tris-buffered saline Tween-20 pH 8.0 (TBST) then 70% methanol and then 95% methanol with 0.3% H_2_O_2_ for 10 min each and 100% methanol for 20 min followed by rehydration. A monoclonal antibody raised against NDUFB8 (a structural component of mitochondrial complex I (complex I-20)) were purchased from Abcam (UK) and used at a concentration of 1:400. The Menapath X-Cell Plus HRP Polymer detection system (A. Menarini Diagnostics, Wokingham, UK) was used for primary antibody detection without a blocking step as per manufacturing guidelines. DAB was added and the sections were dehydrated in grades of ethanol 70%, 95% and 100% twice. Then Histoclear was applied before sections were mounted in DPX®. The degree of immunostaining was then quantified using densitometry.

### 2.5 Fibre typing

Cryostat sections (10 μm) were air dried for 30 min and ATPase activities determined at either pH 4.3 or 4.6 to determine fibre type [10].

### 2.6 Real-time PCR

A quantitative real-time PCR approach was used to calculate both the percentage level of deleted mtDNA and total mtDNA copy number. Fresh frozen muscle sections (20 μm) were mounted on PEN (polyethylenenaphthalate) slides (Leica Microsystems, Milton Keynes, UK) and subjected to sequential COX/SDH histochemistry as described above or SDH staining only, then air-dried after dehydration. Specific muscle fibres were isolated from the section using a Leica Laser Microdissection (AS-LMD) system. The fibres were digested in proteinase K in lysis buffer containing Tris–HCl and 1% Tween, and amplified using PCR primers and fluorogenic probes (Applied Biosystems, Warrington, UK) for regions of *MTND1* (which is highly conserved) and *MTND4* (which is commonly deleted) as previously described [11–13].

### 2.7 Statistical analyses

All data were analysed statistically using Graph Pad Prism® 5 software. Unpaired *t*-tests were used to compare the differences in densitometry between COX-normal Type 2 fibres and COX-intermediate Type 1 fibres at the 60 min time point, the amount of intermediate fibres within each group of patients with differing mtDNA defects and to compare differences in percentage mtDNA deletion level, *MTND1* and *MTND4* copy number per unit volume between the different COX fibre types as well as to compare the calculated values with observed fibre type COX status. Paired *t*-tests were used to compare muscle fibre copy number in fibres reacted sequential for COX/SDH activity and SDH activity alone.

## 3 Results

### 3.1 The COX-intermediate reacting fibre

**Fig. 1:**
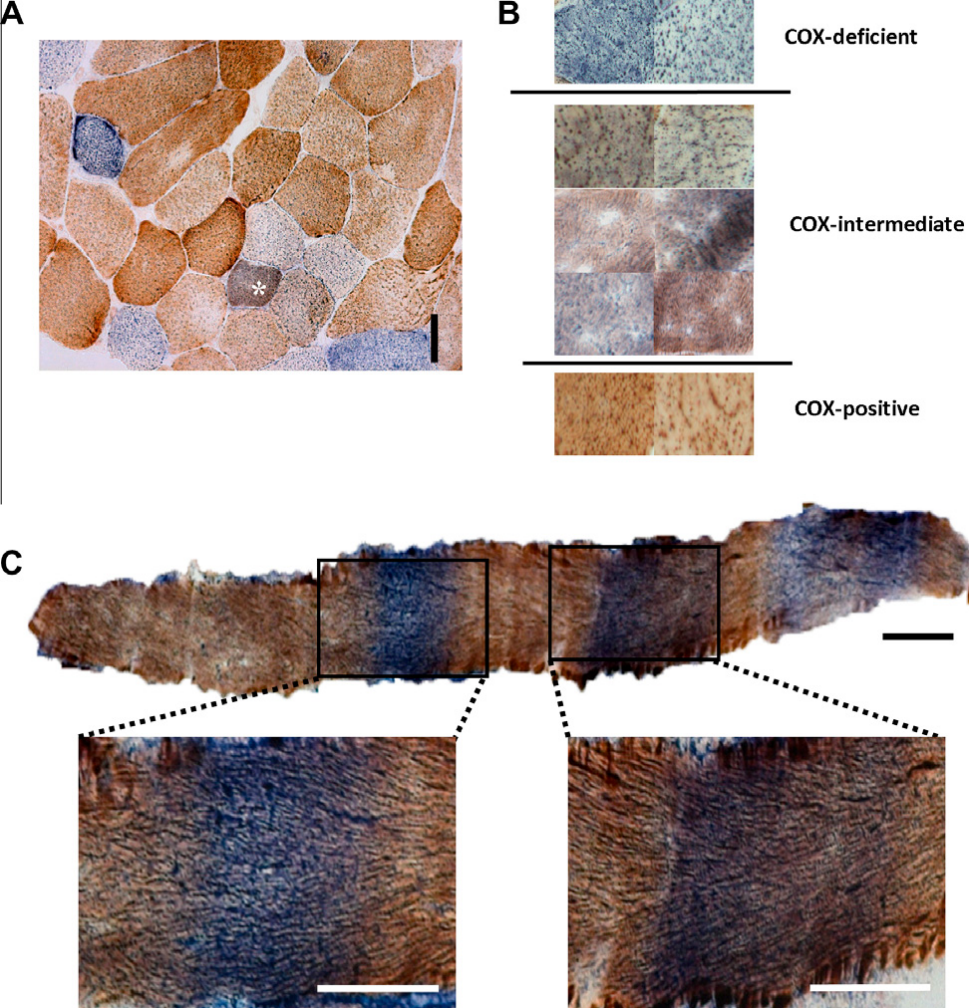
Differences in COX activity differences in individual muscle fibres. (A) Sequential COX/SDH histochemistry of muscle section from a patient with the m.15967G>A mtDNA mutation highlighting an example of an intermediate staining fibre (denoted by asterisk). Scale bar = 50 μm. (B) Illustrative muscle biopsy images from a patient with a single, large-scale mtDNA deletion showing the variation of reaction products observed in individual fibres following sequential COX/SDH histochemistry. (C) A longitudinal muscle fibre from a patient with multiple mtDNA deletions reacted for COX/SDH activities, highlighting the segmental nature of COX deficiency and the COX-intermediate transition zones between COX-deficient and COX-normal segments. Scale bar = 50 μm.

COX activity is demonstrated by an increase in the brown, insoluble indamine polymer, a product of oxidative polymerisation and cyclisation of DAB at the level of cytochrome *c* [14]. Following incubation in the COX reaction medium, an inactive subunit of COX or a defect of the mitochondrial genome resulting in decreased COX synthesis (mtDNA deletion or mt-tRNA mutation) leads to an absence of COX reaction product and this fibre is subsequently termed COX-deficient; when the fibre shows normal activity, it appears brown and is termed COX-normal. Sequential determination of SDH (which is entirely nuclear encoded) activity leads to the formation of a blue reaction product in COX-deficient fibres due to the reduction of NBT by SDH. As the positive staining for COX activity prevents the precipitation of the NBT product in fibres which are already COX-normal, the sequential demonstration of COX/SDH activity is helpful to identify COX-deficient, COX-normal and COX-intermediate reacting fibres [7]. Interestingly, 30 of the 31 patient samples showed fibres that appeared to be neither fully COX-normal nor fully COX-deficient following sequential COX/SDH histochemistry. We subjectively determined these as COX-intermediate reacting fibres (Fig. 1a). There was marked variation within these intermediate fibres as regards the COX/SDH reactivity with fibres appearing purple or grey rather than distinctly brown or blue (Fig. 1b). COX deficiency is segmental along a muscle fibre with the segments ranging from a few to several hundred microns [15]. The COX-intermediate zones will, in part, represent the transitional zone between COX-normal and COX-deficient fibres (Fig. 1c) but there is also variation in the intensity of reactivity between different areas of the same fibre (Fig. 1c).

### 3.2 Investigating COX activity reaction rates

**Fig. 2:**
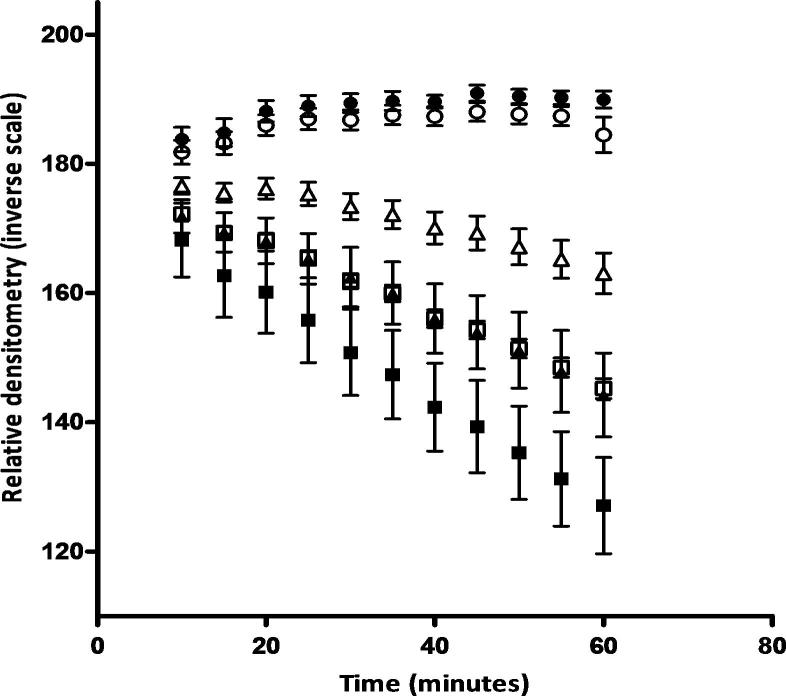
Reaction rates of different fibre types as determined by time lapse densitometry of the COX reactivity. In order to assess if the reaction rate differences were due to fibre type differences, the reaction rates were sub grouped into Type 1 (solid symbols) and Type 2 (open symbols) fibre types. COX-normal fibres are denoted by squares, COX-intermediate fibres denoted by triangles and COX-deficient fibres denoted by circles (mean ± SD values shown). There appears to be a difference in reaction rates between the individual groups and their corresponding fibre type sub group.

In order to assess if COX-intermediate fibres had measurable COX activities that differed from true COX-normal and COX-deficient cells, we determined COX reaction rates for these three groups of fibres within a patient with a single, large-scale mtDNA deletion (Fig. 2). Since the rate of enzyme activity will inevitably be affected by the mitochondrial content of a particular muscle fibre, we also compared activities in both Type 1 (oxidative) and Type 2 (glycolytic) fibres as determined by serial ATPase staining. In total, 73 fibres were reacted three times and an average densitometry reading for each fibre over each time point taken and the average densitometry for each of the individual groups time points compared to each other (these 73 fibres consisted of 17 COX-normal Type 1 fibres, 16 COX-normal Type 2 fibres, 5 COX-intermediate Type 1 fibres, 19 COX-intermediate Type 2 fibres, 6 COX-deficient Type 1 fibres and 10 COX-deficient Type 2 fibres). We showed that for COX-deficient, intermediate and normal fibres as determined subjectively (irrespective of fibre type as determined by ATPase staining), there was an observable difference between the reaction rates, suggesting that the COX-intermediate reacting fibres do represent a distinct population of fibres (Fig. 2). However, we found that using COX activity alone was not sufficient to differentiate between a COX-normal Type 2 fibre and a COX-intermediate Type 1 fibre (Fig. 2). For example, when we compare the differences in densitometry at the 60 min time point between COX-normal Type 2 fibres (145.2 ± 6.158) and a COX-intermediate Type 1 fibres (144.2 ± 14.48) using an unpaired *t*-test, we find that the differences are not significant (*P* = 0.827).

### 3.3 COX-intermediate fibre prevalence within different mitochondrial myopathies

**Fig. 3:**
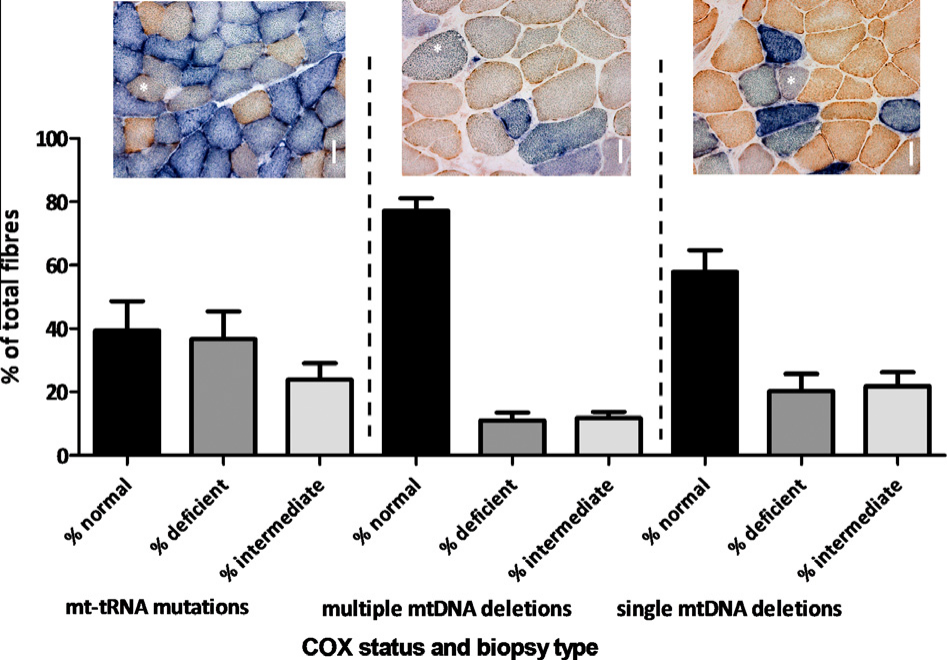
Distribution of COX reactivity in individual fibres from patients with different mtDNA defects Patients were grouped according to their mtDNA molecular defect (*n* = 10 within each group) and the number of COX-normal, COX-deficient and COX-intermediate fibres estimated within biopsies reacted for sequential COX/SDH activities (mean ± SEM). Shown are representative biopsies from patients with the m.5650G>A for the mt-tRNA mutation group, a p.Ala475Gly *PEO1* mutation for the multiple mtDNA deletion group and the 4977-bp common mtDNA deletion for the single, large-scale mtDNA deletion group. Scale bar = 50 μm.

Further analysis of 30 patients with both primary and secondary mtDNA defects revealed that intermediate fibres are present in muscle biopsies from patients with varying mtDNA mutations, who were not undergoing any form of specific exercise training as illustrated in Fig. 3. The incidence of these subjectively observed fibres varied within the groups (mt-tRNA patients 23.89 ± 16.32% intermediate fibres within 3016 fibres counted from 10 patients; single, large-scale mtDNA deletion patients 20.32 ± 16.96% within 2899 of fibres counted from 10 patients; multiple deletion patients 11.76 ± 6.06% within the 4407 fibres counted from 10 patients). Whilst there may seem to be a difference between patients with primary mtDNA defects compared to those with multiple mtDNA deletions, the patients with multiple mtDNA deletions had much fewer respiratory deficient fibres and this is likely to be the major factor in the differences.

### 3.4 MtDNA differences between COX fibre types

Given our particular interest in evaluating the effects of exercise in patients with single, large-scale mtDNA deletions, we wished to investigate any molecular differences between COX-normal, COX-intermediate and COX-deficient muscle fibres. Individual muscle fibres were isolated by laser microdissection from serial sections on PEN slides which had been reacted for either COX/SDH activity or SDH activity alone, prior to the investigation of mtDNA deletion load and mtDNA copy number by real-time PCR. When studying the same fibres which had been reacted for sequential COX/SDH activity and SDH activity alone, we observed using a paired *t*-test, a significantly lower total mtDNA copy number for COX/SDH reacted muscle fibres (9.48 ± 13.62 (mean ± SD) for *MTND1 (total mtDNA)*; 7.72 ± 11.39 for *MTND4 (wild-type mtDNA*) compared to just SDH stained muscle fibres (21.40 ± 8.85 for ND1 (*P* < 0.003); 17.96 ± 8.82 for ND4 (*P* < 0.003)) in the COX-normal fibres (*n* = 12). For COX deficient muscle fibres (*n* = 9) the copy number for COX/SDH stained muscle fibres (17.73 ± 24.51 for ND1; 0.80 ± 0.70 for ND4) was not significantly different when a paired *t*-test was applied to the same cell stained for SDH only (20.53 ± 18.48 for ND1 (*P* = 0.92); and 1.09 ± 1.20 for ND4 (*P* = 0.22)). Together these data suggest that the deposition of DAB in the COX reaction may be affecting the real-time PCR assay used to determine mtDNA copy number. The mechanism of this is uncertain but may be due to the reactive nature of DAB within the mitochondria potentially damaging mtDNA or preventing binding of the PCR primers specific to this real-time PCR assay.

**Fig. 4:**
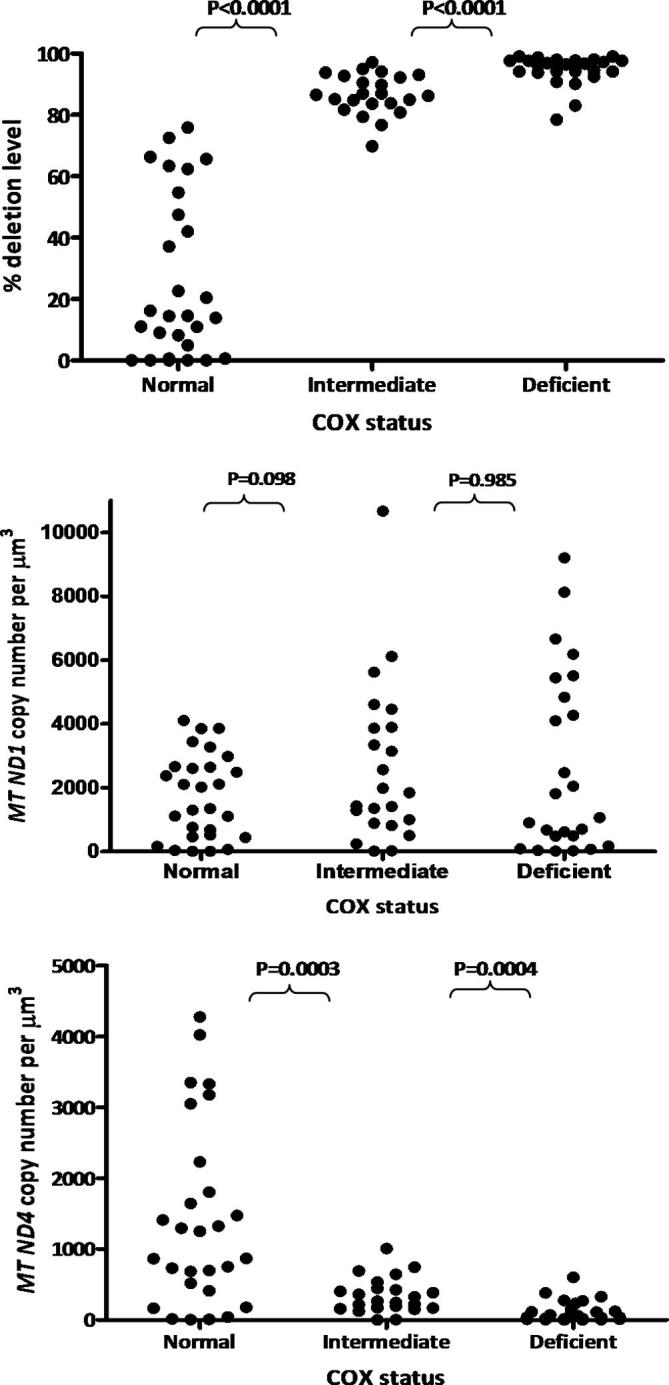
Real-time PCR assessments of mtDNA deletion levels and mtDNA copy number in single fibres from patients with single, large-scale mtDNA deletions. Assays were performed on sections subjected to SDH reaction only for the reasons discussed in the text. (A) distribution of mtDNA deletion load between different fibre categories highlighting that there is a significant difference in the level of mtDNA deletion between COX-intermediate fibres (*n* = 23) and COX-deficient fibres (*n* = 25) (*P* < 0.0001) and between COX normal fibres (*n* = 28) and COX intermediate fibres (*n* = 23) (*P* < 0.0001). (B) Assessment of total mtDNA copy number reveals no significant difference in total mtDNA copy number (*MTND1*) between COX-normal fibres and COX-intermediate fibres or between COX-intermediate and COX-deficient fibers. (C) Assessment of wild-type mtDNA copy number (*MTND4*) indicates highly significant differences between COX-intermediate and COX-normal muscle fibres (*P* < 0.0003) and between COX-intermediate and COX-deficient muscle fibres (*P* < 0.0004).

Due to the observation that the COX reaction product appeared to be affecting the real-time PCR assay, we cut serial sections from patients with single, large-scale mtDNA deletions onto both glass and membrane slides, staining the glass slide for sequential COX/SDH activity to determine the COX status of individual muscle fibres. These fibres were then identified on the membrane slide which was reacted for SDH activity alone to ensure that the real-time PCR was not affected by deposition of the DAB reaction product in the COX reaction (Fig. 4). We showed that there was a significant difference in the percentage level of deleted mtDNA between COX-normal (*n* = 28) and COX-intermediate fibres (*n* = 23) (*p* < 0.0001) and between COX-intermediate fibres and COX-deficient fibres (*n* = 25) (*p* < 0.0001), although we did not observe a significant difference in the total mtDNA copy number per μm^3^ (*MTND1* probe) between COX-normal and COX-intermediate fibres (*p* = 0.098) or between COX-intermediate and COX-deficient fibres (*P* = 0.98). A highly significant difference in the wild type mtDNA copy number per μm^3^ between COX-intermediate reacting and COX-normal muscle fibres (*P* < 0.0003) and between COX-intermediate reacting and COX-deficient muscle fibres (*P* < 0.0004) was noted, providing further evidence that wild-type mtDNA copy number is the critical determining factor in COX activity status.

### 3.5 Further characterisation of the intermediate fibre

**Fig. 5:**
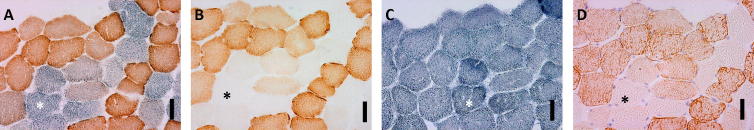
Histochemical and immunohistochemical assessment of mitochondrial function in a patient with a single, large-scale mtDNA deletion. A, B, C, and D show sequential COX/SDH activity, COX activity, SDH activity and CI-20 immunoreactivity respectively in serially-cut sections. Shown is a single COX-deficient muscle fibre (asterisk), highlighting the relationship between COX deficiency as determined with the dual enzyme reaction with both the individual COX reaction and CI-20 immunoreactivity. Scale bar = 50 μm.

We wanted to better define the biochemical characteristics of the COX-intermediate reacting fibres using an objective measure rather than the subjective observation of the colour of the reaction product. In addition to COX (complex IV of the mitochondrial respiratory chain), complexes I, III and V also contain subunits encoded by the mitochondrial genome. Unfortunately it has not been possible to develop histochemical assays to determine the activities of these complexes in tissue sections; however, defects in immunoreactive protein expression can be detected by immunocytochemistry using monoclonal antibodies to specific respiratory chain subunits [16,17]. We chose to investigate complex I since biochemical deficiency of this complex is a common observation in tissues from patients with mtDNA mutations causing defects in mitochondrial protein synthesis due to the large number of subunits of this complex encoded by the mitochondrial genome. In the absence of antibodies to the mitochondrial-encoded subunits of complex I, we used an antibody to the 20-kDa subunit NDUFB8 (CI-20). In the absence of the mitochondrially encoded subunits the assembly of the holocomplex is impaired at an early stage [18] and there is loss of nuclear-encoded mitochondrial subunits including NDUFB8. To determine if we could better define which fibres were respiratory-normal, intermediate and deficient, we used the densitometric measurements for COX, SDH and CI-20 of individual fibres from serial sections. Both COX and complex I contain key catalytic subunits encoded by the mitochondrial genome and we believed that both would be low in fibres with high levels of mtDNA mutation; SDH activity was used as a measure of mitochondrial respiratory chain content (thus removing the need to fibre type). The advantage of measuring COX and SDH is that their activities are still linear at the end of the histochemical assay. Illustrative images of staining techniques used are shown in Fig. 5 and the data from these staining methods allowed us to determine the respiratory status of the fibre using the following equation:

$$(\frac{\text{COX densitometry}\times \text{CI-}20\;\text{densitometry}}{\text{SDH densitometry}}/100$$

**Fig. 6:**
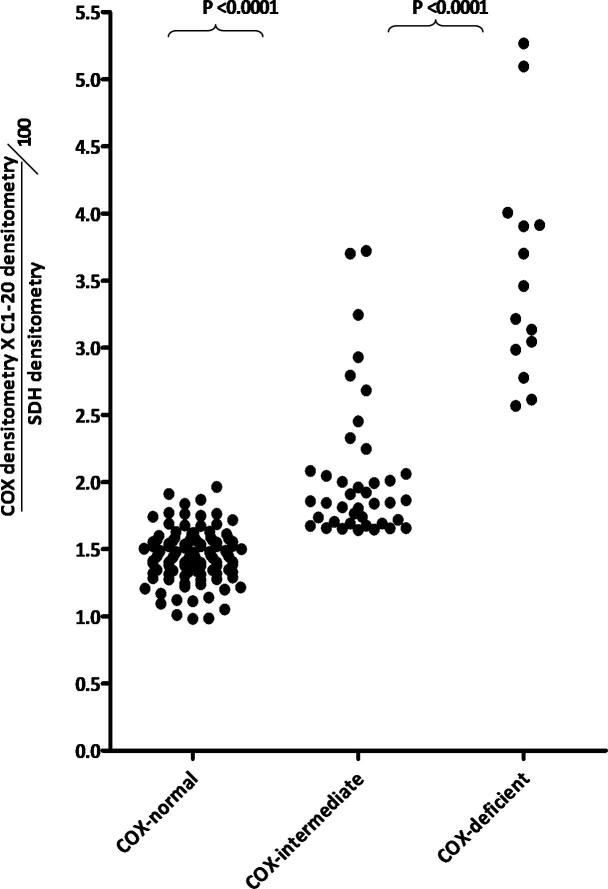
Objective measurements of COX reactivity compared with subjective sequential COX/SDH reactivity. We assessed 172 muscle fibres from a patient with a single, large-scale mtDNA deletion. Significant differences are observed between COX-intermediate and COX-normal muscle fibres (*P* < 0.0001) and between COX-intermediate and COX-deficient muscle fibres (*P* < 0.0001).

**Fig. 7:**
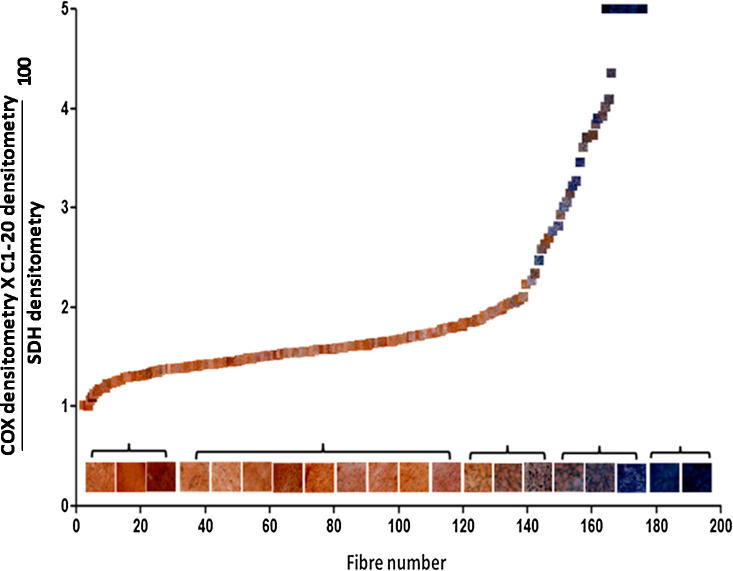
$(\frac{\text{COX densitometry}\times \text{CI-}20\;\text{densitometry}}{\text{SDH densitometry}}/100\;\text{values}$ compared with COX/SDH reactivity. Serial sections (10 μm) of muscle tissue from a patient with single, large-scale mtDNA deletion were reacted for dual COX/SDH activities and individual COX, SDH and CI-20 reactivity in order to determine the biochemical status in 200 fibres. In serial sections the dual COX/SDH reactivity was determined and the images overlaid on the biochemical results for each fibre. Larger representative images are also displayed on the *X* axis. This figure illustrates the normal stoichiometry of respiratory chain complexes as shown by the initial line, followed by the gradual and then steep increase in this value as a function of the progressive loss of complex I and COX whilst the SDH is maintained and then increases. The final few values represent a complete loss of both complex I and COX and maximal over expression of SDH.

We then compared the value we obtained using our objective method with the subjective observations. We compared the values obtained for individual fibres using this equation and grouped them according to the subjective visualised observation of COX/SDH status in a vertical scatter plot. There is a significant difference in the objective values using the equation which depends on the individual COX status (Fig. 6). In addition the objective method shows the variation seen in the mitochondrial enzyme activity within the intermediate fibre group and allows us to detect subtle changes in enzyme activity (Fig. 7).

## 4 Discussion

Muscle biopsies from patients with mitochondrial myopathies often show a mosaic picture of COX-deficient and normal reacting, COX-normal fibres. The striking blue fibres seen on a combined COX/SDH histochemical stain strongly indicate the presence of a genetic defect involving the mitochondrial genome. Here we show that in addition to segments of muscle fibres with a clear biochemical phenotype, there are often many fibre segments in a diagnostic muscle biopsy which show intermediate COX reactivity, with evidence of partial loss of COX activity. We also demonstrated that within a muscle biopsy from a patient with a single, large-scale mtDNA deletion there was a different genetic profile (at the mtDNA level) for these cells, confirming they are between COX-normal and COX-deficient.

To obtain an objective measure of the respiratory status of individual muscle fibres to a mitochondrial genetic defect, we have used the densitometric changes seen in complex I (protein expression) and COX (enzyme activity), both of which would be affected by a generalised defect in mitochondrial protein synthesis due to a single, large-scale mtDNA deletion or an mt-tRNA mutation, together with the activity of SDH (complex II) within the muscle fibre. The advantage of this approach is that it quantifies the progressive alteration in the stoichiometry of respiratory chain complexes that occurs as a consequence of the severity of the molecular defect. Using this objective measure we not only observe a clear difference between COX-normal reacting, COX-intermediate and COX-deficient muscle fibres, but also document a gradation of the biochemical defect in COX activity in COX-intermediate fibres. This confirms our theory that these fibres represent an intermediary stage between COX-normal and COX-deficient fibres.

An unexpected finding during these studies was the observation that the DAB reaction product within the COX histochemical reaction had an adverse effect on the efficiency of our real- time PCR assay for mtDNA copy number, as shown by the significant difference between sections stained for SDH activity alone versus those in which had been reacted for sequential COX/SDH activities. Interestingly, the effect was not seen in totally COX-deficient cells due to the lack of the DAB COX reaction product in these cells. This finding has significant consequences for studies in which the molecular mechanisms of COX deficiency are being explored, in particular when looking to determine the absolute amount of wild-type or mutated mtDNA copy number [19]. For diagnostic studies in which this assay is used to quantify mtDNA deletion levels [6] this does not appear to be an issue since the effect seems to be the same on both templates.

Why might the characterisation of COX-intermediate reacting fibres be important? In terms of making a diagnosis in patients with suspected mitochondrial myopathy, the presence of clearly detectable, blue-staining COX-deficient, SDH-positive fibres remains a very robust measure for detecting the presence of a respiratory chain defect [6]. However, when we consider the use of muscle biopsies in the evaluation of clinical studies, such as response to exercise training protocols or in terms of disease progression, then small and subtle changes in the respiratory activity of individual muscle fibre segments, themselves reflecting subtle changes in either mtDNA mutation load or mtDNA copy number, will not be detected by the histochemical techniques currently employed. Measuring mitochondrial respiratory chain enzyme activities in tissue homogenates offers one approach, but the individual complex assays are challenging to perform and are not sensitive enough to detect small overall changes in activity. It is worth noting that patients with mosaic COX defects can demonstrate normal muscle respiratory chain enzyme activities simply because of the relatively small number of COX-deficient fibres in some muscle biopsies [20]. If, as we believe, the COX-intermediate reacting fibre segments are an important transitional stage from normal enzyme activity status to COX-deficient, and vice versa, therefore an increase in the number of these transition zones indicates that these fibre segments have elongated or COX activity status has changed. It is crucial that these are accurately documented in different biopsies and this can only be done using the methods proposed.

The link between the respiratory chain activity and mtDNA status was further explored in individual muscle fibres from a patient with a single, large-scale mtDNA deletion. We were able to show a different genetic profile for each fibre type but the defining factor was the level of wild type mtDNA, not the level of deleted mtDNA. When describing a threshold for respiratory chain deficiency, we often discuss this in terms of levels of mutated mtDNA but these data and those from other studies confirm the importance of the amount of wild type mtDNA in determining the biochemical phenotype [21]. It is therefore clear that subtle changes in the copy number of wild type mtDNA could have major effects on the biochemical properties of individual muscle cells. Thus strategies to increase mtDNA copy number such as endurance exercise are likely to be effective in improving muscle function [22,23].

In conclusion, we have explored the respiratory chain phenotype in individual muscle fibres from patients with mtDNA disease and shown that COX-intermediate reacting fibres are commonly observed in association with a variety of different mitochondrial genetic defects. These fibres represent a distinct population of cells with levels of activity which are between those seen in fully COX-normal reacting and COX-deficient fibres, and therefore represent a potentially important phenotype to assess if accurate measures of mitochondrial disease progression or the positive effects of treatment on mtDNA mutation levels and biochemical activities are to be investigated. We show this is possible using a combination of histochemical and immunocytochemical techniques and are currently applying this practice to our ongoing studies.
